# Influence of the Charge Stoichiometry on the Properties of Biopolymer Films Based on a Polyelectrolyte Complex of Chitosan and Carboxymethyl Starch

**DOI:** 10.3390/polym17243293

**Published:** 2025-12-12

**Authors:** David Castro, Valentina Brovina, Mikhail Litvinov, Aleksandr Podshivalov, Lucía Castro, Diana Chamorro, Omar Oña, Adriana Tapia

**Affiliations:** 1 Center for Chemical Engineering, ITMO University, Kronverkskiy Prospekt, 49, 197101 Saint-Petersburg, Russia; dcastrovargas@itmo.ru (D.C.); vbrovinaa@itmo.ru (V.B.); mikhail.litvinov.1996@mail.ru (M.L.); 2 Departamento de Ciencias Exactas, Universidad de las Fuerzas Armadas ESPE, Sangolquí P.O. Box 16 171-5-231B, Ecuador; lecastro4@espe.edu.ec (L.C.); dcchamorro@espe.edu.ec (D.C.); orona2@espe.edu.ec (O.O.); aetapia6@espe.edu.ec (A.T.)

**Keywords:** chitosan, carboxymethyl starch, polyelectrolyte complex, films

## Abstract

Biopolymeric films based on chitosan and starch offer an ecological alternative for food protection. Nevertheless, their practical application is often limited by their low mechanical properties and high solubility in aqueous solutions, due to weak interactions between the chains of the biopolymers. One approach to resolve this problem is to obtain biopolymeric films based on (bio)polyelectrolyte complex ((bio)PEC). These films exhibit stronger electrostatic interactions and homogeneous biopolymeric structure. In this study, films based on (bio)PEC were obtained by the casting method, using chitosan and carboxymethyl cassava starch with different degrees of substitution with a biopolymer concentration of 2.5 wt.% at pH = 6. The obtained films were analyzed using the optical and scanning microscopy, color method, ATR-FTIR spectroscopy, thermogravimetry, mechanical analysis under tension, solubility in water, simulated gastric fluid (SGF), and phosphate-buffered saline (PBS) solutions, and contact angle of water. The results demonstrated that the tensile strength and Young’s modulus of films based on (bio)PEC increased by 2–4 times, and the elongation at break by 20% compared to films based on a mixture chitosan and starch. This is due to the increase in the attraction between oppositely charged polyelectrolytes in (bio)PEC films. Additionally, the solubility of (bio)PEC films was reduced by ~40%, 35% and 70% in water, SGF and PBS solutions, respectively, when the carboxymethyl starch with highest degree of substitution was used, and *z* was near to 1.

## 1. Introduction

Recently, there has been great interest in the development of biopolymeric edible films [1]. Product storage in these films due to their unique membrane characteristics is accompanied by proper gas and vapor transfer between the environment and the fruit, allowing the product to “breathe” properly. This contributes to longer freshness of the produce by slowing down oxidation processes, moisture loss, enzymatic activity, and microbial spoilage [2,3,4]. Such films are ideal for storing meat, fruits and vegetables, effectively extending their shelf life [5].

**Figure 1:**
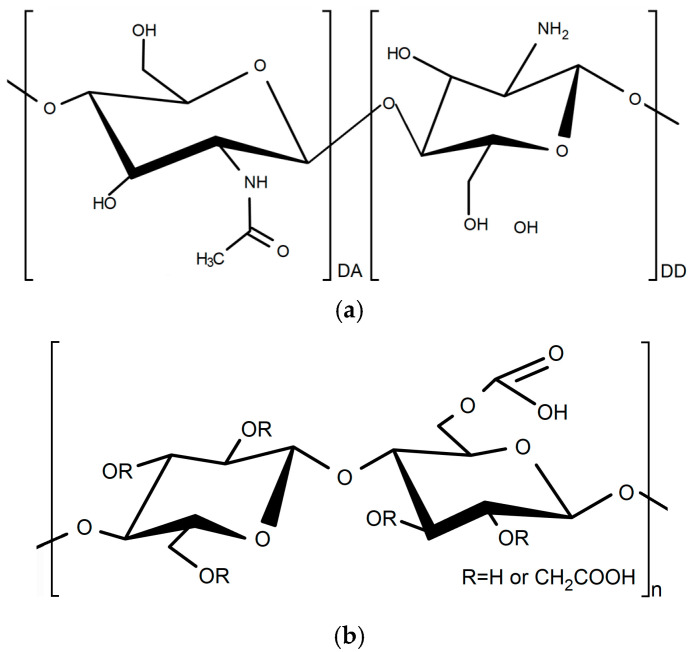
Chemical structure of (**a**) chitosan and (**b**) carboxymethyl starch.

Edible films are used in various food packaging applications and are valued for their physical properties, biochemical stability and non-toxicity [6]. Most of the starting materials used to create edible packages are derived from natural renewable resources such as polysaccharides, proteins, lipids and their composites [7,8]. Among all available biopolymers, cassava starch and chitosan are prominent as a widely used edible packaging material made from polysaccharides [9,10]. The attractiveness of these polymers is their low cost, variety of available raw materials and enhanced biological activity [11,12,13,14]. Cassava starch is a common biopolymer derived from the root of the perennial cassava plant Manihot esculenta grown in tropical regions. It is the third-largest source of carbohydrates among vegetables and one of the most important crops in the world [15,16]. Starch shows potential for sustainable food packaging because it is an abundant, cheap, biodegradable, biocompatible, renewable and edible biopolymer. However, starch films have limitations attributable to their low mechanical strength and high sensitivity to moisture [17]. Chitosan is a linear polysaccharide with a structure composed of repeating units of D-glucosamine and *N*-acetyl-*D*-glucosamine, linked by β-(1→4) glycosidic bonds (Figure 1a). Chitosan is a widely available natural, non-toxic, biodegradable and common cationic polysaccharide derived from the deacetylation of chitin. Chitosan has enhanced antimicrobial properties due to its polycationic characteristics [18,19]. Some works have prepared and studied the use of different chitosan-based edible films used as food packaging and covering. Edible films based on chitosan and starch can act as active packaging, thereby extending the shelf life of food products, as a result of their antioxidant and antimicrobial activity [20,21].

Despite the advantages of films based on chitosan and starch, it has been previously shown that films made from individual biopolymers or from a combination of these polysaccharides often have low chemical and thermal stability, as well as reduced mechanical strength, which limits their application in the packaging industry [22]. In addition, due to their high content of hydroxyl, amino or carboxyl groups, they exhibit pronounced hydrophilicity, which impairs their barrier properties against water vapor and moisture [23]. One option to address this problem is the development of materials based on (bio)polyelectrolyte complexes ((bio)PEC). A polyelectrolyte complex is a structure formed when oppositely charged polyelectrolytes are mixed in solution. The basic strategy for obtaining (bio)PEC involves mixing the individual polyelectrolytes while adjusting the pH of the medium to achieve the ionization of oppositely charged functional groups. This process leads to the self-assembly of the components into complex supramolecular structures [24].

One of the biopolymers used for obtaining (bio)PEC, with chitosan, is carboxymethyl starch (CMS). Carboxymethyl starch is an ether derivate of starch product, synthesized by a carboxymethylation reaction between activated starch, with sodium hydroxide, and monochloroacetic acid, where hydroxyl groups (OH) are replaced by carboxymethyl groups (CH_2_COO). The fundamental structure of CMS is inherited from native starch, a carbohydrate polymer composed of *D*-glucose units linked primarily by α-1,4 glycosidic bonds (Figure 1b) [25,26].

In recent years, the preparation of (bio)polyelectrolyte complexes ((bio)PEC) based on polysaccharides, like chitosan and CMS, has been a challenging task due to the many variables associated with the monosaccharide interrelationships and structural regularity. Additionally, the balance of molecular interactions during complexation is significantly influenced by external factors such as pH, ionic strength, and temperature [27]. Materials from (bio)PEC, based on polysaccharides, are gaining importance for their potential in various applications, like medicine, food packaging, and smart materials, because of their unique properties [24,28,29]. Nanoparticles, hydrogels and films can be obtained from (bio)PEC based on chitosan and CMS. For example, Quadrado R. and Fajardo A. [30] demonstrated that the microparticles based on (bio)PEC from CMS and chitosan, with a ratio of 3:4 Chit/CMS, respectively, showed higher chemical and thermal stability in comparison with the particles obtained from conventional ionotropic crosslinking of chitosan and sodium tripolyphosphate (TPP). Furthermore, the microparticles of the CMS/Cs polyelectrolyte complex demonstrated a better encapsulation of bovine serum albumin (BSA) and confer an ideal pH-dependent release profile. This makes them promising systems for use in the field of drug delivery in the gastrointestinal tract.

In another study [31], a nanocomposite of chitosan and carboxymethyl starch was developed, with a ratio of 3 to 1, respectively, and 1 wt.% of montmorillonite (MMT) was added for the release of curcumin. The interaction between the Chit and the CMS was fundamental for obtaining a suitable particle size for the encapsulation and release of the active agent. The addition of MMT enhanced the curcumin encapsulation efficiency to reach 91% in the optimal formulation, which had an average particle size of 35.9 nm. The biocomposite loaded with curcumin effectively inhibited the formation of *Streptococcus mutans*, demonstrating its potential application in the dental field. In the work of Henao E. et al. [32] the polyelectrolyte complexation and ionotropic gelation methods were compared by the obtention of hydrogels based on Chit and CMS with polymer ration of 16:1, respectively. The author concluded that the particles obtained by polyelectrolyte complexation tend to agglomerate.

The stoichiometric charge of polyelectrolyte complexes (*z*) is a critical parameter defining their properties. This charge represents the ratio between the number of positively and negatively charged monomers forming the (bio)PEC. A representative example of how this parameter influences material properties is described in the study [33]. Hydrogels prepared from high molecular weight chitosan and gelatin with different z values demonstrated that materials with *z* ≈ 1 achieved an elastic modulus four times greater than pure gelatin hydrogels. Additionally, these materials showed increased sorption capacity and enhanced stability in aqueous solutions. These results indicate that the material properties are optimized when *z* approaches 1. In their study, Boughamin et al. [34] also investigated the influence of the charge ratio in (bio)PEC based on poly(ethylene alt-maleic acid) (PEMA) and chitosan with different molecular weights. The results showed that particle sizes of approximately 200 nm, initially observed at a charge ratio of 0.2, increased fourfold as the PEMA ratio increased, reaching a maximum near a 1:1 ratio of polyelectrolytes. Additionally, the study revealed that the negatively charged (bio)PECs exhibited larger particle sizes compared to their positively charged counterparts. In another study [35], the development of films with different charge ratios, based on the use of a volatile base, was investigated. The polyelectrolytes used were poly(acrylic acid) (PAA) and polyethylenimine (PEI), and oxygen permeation was analyzed. The results showed that films based on (bio)PEC with charge ratios *z* > 1 are much less sensitive to water compared to the pure polyelectrolytes. Whereas, in films with *z* < 1, the permeability is lower. This study concluded that the charge ratio allows for the adjustment of properties such as gas barrier and mechanical resistance.

The analysis of the literature shows that most of the studies on (bio)PEC based on chitosan and carboxymethyl starch are aimed at obtaining hydrogels and dispersions. Similarly, the influence of the stoichiometric charge ratio has been analyzed in different systems and properties, such as particle size in solutions, mechanical properties in hydrogels and barrier properties in films. However, the influence of the charge ratio on the mechanical properties of (bio)PEC films has been insufficiently studied. In this respect, the main objective of the present study was to study the influence of the ratio of ionized groups *z* of (bio)PEC on the physico-mechanical characteristics of (bio)PEC-based films from carboxymethyl starch (CMS) with different degree of substitution, and chitosan by a casting method. For this purpose, (bio)PEC dispersions were prepared by mixing solutions of CMS and adjusting the pH. The obtained films were compared with films based on chitosan and starch, and all the films were characterized by color surface method, optical and scanning microscopy, ATR-FTIR spectroscopy, and thermal analysis techniques. The improvement in the mechanical properties of (bio)PEC films, as well as their reduction in the percentage of solubility, was demonstrated.

## 2. Materials and Methods

### 2.1. Materials

Cassava starch (Thai Food King, Samutprakarn, Thailand), and chitosan with molecular weight of 30 kDa and degree of deacetylation (DD) of 0.82 obtained from crustacean shells (crabs, shrimp) (Bioprogress, Biokombinata, Russia) were used as biopolymers. Sorbitol (Sigma-Aldrich, Saint Louis, MO, USA) was used as a plasticizer. Distilled water was used as a solvent. Solutions of NaOH (0.1 M) and HCl (0.1 M) were used to set the pH. Carboxymethyl starches and their degree of substitution (DS) were obtained and determined, respectively, following the method of our previous study [36]. Three products were obtained by a two-step reaction process of carboxymethylation: alkalization followed by etherification. Starch was treated with NaOH and different amounts of monochloroacetic (MCA) (2, 2.5 and 3 g) at 50 °C for 3 h. The products were sedimented with isopropanol for 24 h at room temperature and dialysis was carried out to purify the products. The obtained products were named CMS 1, CMS 2, and CMS 3 with different DS values: 0.16, 0.33 and 0.36, respectively. The DS values were determined by direct titration method. For this purpose, the products of CMS were dispersed in NaCl solution (2 wt.%) and titrated with a solution of NaOH (1 M) [36,37].

### 2.2. Preparation of Chitosan and Carboxymethyl Starch (Bio)PEC Films

First, to confirm the formation of the polyelectrolyte complexes, and to determining the pH range of its formation, the turbidimetric titration was used. For this purpose, chitosan and CMS individual solutions were prepared with a concentration of 0.25 wt.% at temperatures of 25 and 40 °C, respectively. The chitosan and CMS solutions were mixed with a 1:3 *v*/*v* ratio and titrated in a pH range from 2 to 12 by adding 0.1 M NaOH and HCL aliquots. The transmittance of obtained solutions was measured with a spectrophotometer (Unico, Franksville, WI, USA) at a constant wavelength of λ = 364 nm at 25 °C. From these results the pH value for the preparation of the (bio)PEC films was chosen. The pH selection was based on the principle of obtaining the lowest transmittance, i.e., highest of polyelectrolyte complex volume is formed for mixtures with all CMS products [36].

The films of a mixture of chitosan and CMS products with different DS were obtained by casting a film-forming solution at different ratio of weight. For this purpose, the individual solutions of polymers with a concentration of 2.5 wt.% were prepared by dissolving them in distilled water with constant stirring at a temperature of 40 °C for chitosan solution and 25 °C for CMS solutions for 1 h. The constant temperature with an accuracy of ±0.2 °C was maintained using a digital thermostatic thermometer ETS–D6 (IKAWerke, Staufen, Germany). After biopolymer dissolving, a film-forming solutions of chitosan and CMS (named as Chit/CMS) were prepared by mixing them with a ratio of 1:3 *v*/*v*, and the pH of the mixtures was measured and adjusted until pH = 6 with a solution of HCl (0.1 M). This pH and polymer ratio was used for obtaining polyelectrolyte complex dispersions of (bio)PEC with the value of the ratio of ionized groups (*z*) close to 1, according to the results of turbidimetric titration. The ratio of ionized groups *z* reflects the process of polyelectrolyte interaction and depends on the content of ionized functional side groups in the individual biopolymers. This approach is based on the condition that the degree of ionization of functional groups in both polyelectrolytes is close to 1. The values of *z* were calculated using Equation (1) [36].

(1) $$z=\frac{\left[{COO}^{-}\right]}{\left[{NH}_{3}^{+}\right]}$$

where [COO^−^] and [NH_3_^+^] are the molar concentration of charged units. The *DD* of chitosan and DS values of CMS were considered for calculating the values of *z.* The values of *z* were 0.30, 0.64 and 0.98 for Chit/CMS 1, Chit/CMS 2, and Chit/CMS 3, respectively.

After the pH adjust, sorbitol in 20 wt.% from the dry mass of biopolymers was added to each dispersion of (bio)PEC Chit/CMS and mixed at a constant temperature of 60 °C for 30 min. Then, the casting technique was used to prepare the films. For this, a volume of 15 mL of the prepared dispersions was molded to Petri dishes with a diameter of 90 mm, followed by drying in a laboratory oven at 35 °C with constant air convection for 24 h. The process described above, without pH control, was replicated to obtain biopolymeric films based on chitosan and native cassava starch without carboxymethylation (named as Chit/Starch).

### 2.3. Characterization of Films

#### 2.3.1. Color Properties and Microscopy

Color properties in the system of CIE L* (lightness), a* (negative green; positive yellow), and b* (negative blue; positive yellow) were determined by measuring the dependence of reflectance from wavelength. To convert the spectrum to color properties, first, the CIE X, Y, Z tristimulus values were determined from the spectral data using the CIE formula [38,39]. Then, color space transformation formulas (Equation (2)) were used to convert these X, Y, Z values into CIE L*a*b* coordinates [38,40]. The results were calculated by the Excel spreadsheet by Bruce Justin Lindbloom [38,41,42]. A spectrophotometer UV-1800 (Shimadzu, Kyoto, Japan) was used to conduct this experiment. Measurements were carried out in the wavelength range from 340 to 830 nm and observed at a 2° angle. All measurements were performed seven times on different areas of films.

(2) $$\begin{array}{c} L^{*}=116\;f\left(Y\right)-16\\ a^{*}=500[\;f\left(X\right)-f\left(Y\right)]\\ b^{*}=200[\;f\left(Y\right)-f\left(Z\right)] \end{array}$$

where L*, a* and b* are the three coordinates of the CIE L*a*b* model; $f\left(X\right)$, $f\left(Y\right)$ and $f\left(Z\right)$ are defined in Equation (3):

(3) $$\;f\left(j\right)=\left\{\begin{array}{c} \;\;\;\;\;\;\;\;\;\;\;\;\;\;\;\;\;\;\;\;\;\;\;\;\;\;\;\sqrt[{3}]{\frac{j}{j_{n}}}\;\;\;\;\;\;\;if\;\frac{j}{j_{n}}>0.008856\\ \frac{903.3\;\frac{j}{j_{n}}+16}{116}\;\;\;\;\;\;\mathrm{otherwise} \end{array}\right.,$$

where *j* represents the value *X*, *Y* and *Z*, in the coordinates in the model CIE XYZ; and *j_n_* are the values *X_n_*, *Y_n_*, and *Z_n_* for the refence white.

In addition, for analyzing the morphology of the films, an optical microscope in transmission mode STM6 (Olympus, Tokyo, Japan) and a scanning electronic microscope Mira III (Tescan, Brno, Czech Republic) working at 2.0 kV were used.

#### 2.3.2. Attenuated Total Reflectance Fourier-Transform Infrared Spectroscopy Analysis of Films

The chemical composition of the obtained films was analyzed using ATR-FTIR spectroscopy. Spectra were recorded using a Tensor 37 spectrometer (Bruker, Karlsruhe, Germany) equipped with a ZnSe crystal ATR unit (MIRacle, Pike, WI, USA), covering wavenumber ranges of 4000–500 cm^−1^ with an increment of 2 cm^−1^, averaged over 32 measurements.

#### 2.3.3. Thermal Analysis of Films

Thermal properties were studied by thermogravimetric analysis (TGA) and differential scanning calorimetry (DSC).

The thermal stability of the films was evaluated using a 209 F1 Libra thermomicrobalance (Netzsch, Selb, Germany). Experiments were performed in a dry nitrogen atmosphere with a controlled heating rate of 10 °C/min, spanning temperatures from 25 to 900 °C. Aluminum oxide (Al_2_O_3_) measuring crucibles served as sample containers during the analysis.

The glass transition temperature (*Tg*) and other endothermal effects of the films were determined using DSC calorimeter 204 F1 Phoenix (Netzsch, Selb, Germany), operating at a heating rate of 10 °C/min in a temperature range from −30 to 280 °C. Before the analysis, samples were heated from room temperature to 150 °C to eliminate the moisture. After that, films were cooled until −30 °C and then heated again to a temperature of 280 °C. The experiments were carried out with nitrogen as purge gas at a flow rate of 50 mL/min. Samples were encapsulated in aluminum crucibles with a target sample weight of 3 mg ± 1 mg. In order to determine *Tg*, the data from the second heating were analyzed.

#### 2.3.4. Mechanical Analysis of Films

The tensile strength (*σ*), Young’s modulus (*E*), and elongation at break (*ε_b_*) were measured using a universal testing machine Instron 5943 (Instron, Norwood, MA, USA). For this purpose, seven samples of each film were evaluated. Film thickness was determined by directly measuring 10 different points for each film with a digital micrometer (Tehrim, Moscow, Russia) with an accuracy of 0.004 mm. Then, film samples were cut in the form of strips with dimensions of 50 × 10 mm and subjected to tensile deformation at a constant rate of 15 mm/min at room temperature.

#### 2.3.5. Solubility Test of Films in Liquids

For this study seven samples for each film composition were cut with dimensions of 3 × 3 cm and dried at 65 °C for 24 h [43]. Then, the dried films were weighed and placed into glass with 80 mL filled with deionized water, phosphate-buffered saline (PBS) solution, and simulated gastric fluid (SGF) solution, separately. The samples were kept at 37 °C with constant stirring for 1 h. After that, the solutions containing films were filtered, the residues were dried at 65 °C and weighed. The percentage of the total weight loss of film was calculated according to Equation (4).

(4) $$Solubility=\frac{m_{o}-m_{f}\;\;}{m_{o}}\times 100\%$$

where *m_o_* and *m_f_* are the initial and final mass of the samples, respectively.

#### 2.3.6. Water Contact Angle of Films

The contact angle of distilled water on obtained films was determined using the sessile drop method with drop shape analyzer DSA 100E (KRÜSS, Hamburg, Germany). All measurements were performed on seven drops of water at different points on the surface of the samples at room temperature.

## 3. Results and Discussion

### 3.1. Effect of the pH on the (Bio)Polyelectrolyte Complex Formation

**Figure 2:**
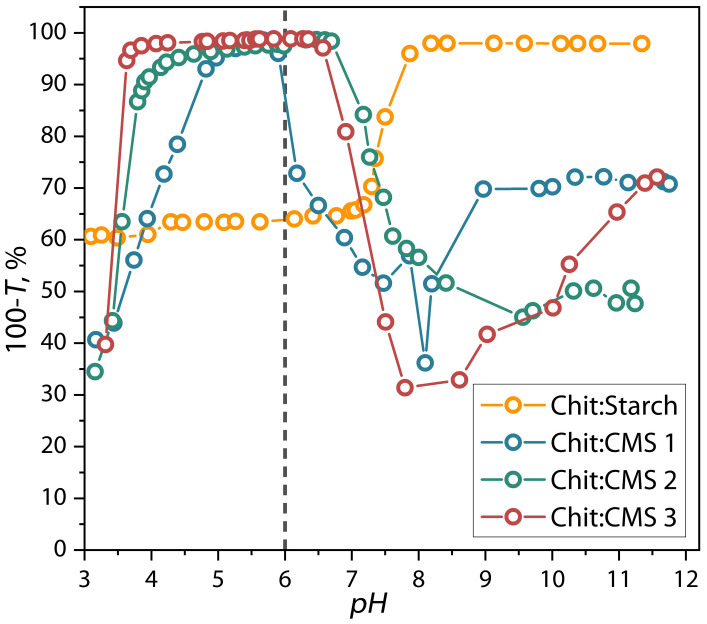
Dependence of reverse transmittance of dispersions of chitosan with CMS 1, CMS 2, and CMS 3 on the pH.

The results of the influence of pH on the formation of (bio)PEC is shown in Figure 2.

As seen in Figure 2 the 100-*T* value for the mixtures strongly depends on pH which demonstrates the formation of the dispersions of (bio)polyelectrolyte complex. Additionally, the results show that the Chit/CMS dispersions can be delimited by four zones of pH changing. The first zone, with a limit of pH < 4, corresponds to the formation of unstable and soluble dispersion with low 100-*T* values. The second zone corresponded to the maximum values of 100-*T* and associated with the formation of insoluble (bio)PEC structures with greater turbidity. In this zone the polyelectrolytic interaction process occurs more effectively and stoichiometric complexation is achieved. For the previous reasons, a pH in this range (pH = 6) was chosen for obtaining the (bio)PEC-based films, ensuring the formation of the dispersions with rich microphase of (bio)PEC. In the third zone, at pH higher than 6.5, a reduction in the 100-*T* is observed, which is associated with the destruction of the (bio)PEC structures due to the deprotonation of the amino groups of the chitosan because the pH values are close to *pK*(chitosan) = 6.34. Finally, for the last zone at pH > *pK*(chitosan), the solution contains insoluble chitosan microphase and dissolved macromolecules of CMS [36,44,45,46].

### 3.2. Color Properties and Visual Aspect of Films

**Figure 3:**
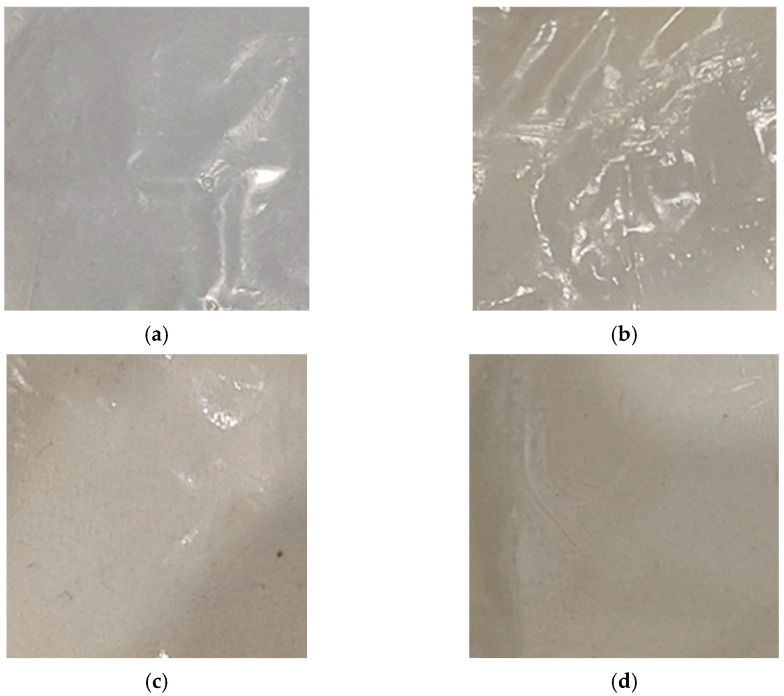
Macrophotographs of (**a**) Chit/Starch, (**b**) Chit/CMS 1, (**c**) Chit/CMS 2 and (**d**) Chit/CMS 3 films.

The color of biopolymeric films has a direct impact on the pursuit and acceptance of consumers [47]. The appearance of the obtained films and the color properties depend on the biopolymer used. The macrophotographs and color properties of the films are shown in Figure 3 and Table 1, respectively.

From Figure 3 it can be seen that the biopolymeric films presented homogeneous surfaces, and absence of insoluble (bio)PEC Chit/CMS particles. Additionally, the values of the color parameters L*, a*, and b* in Table 1 corroborate the differences in lightness appearance between films from (bio)PEC and Chit/Starch. The highest value of L* (lightness) corresponds to the Chit/Starch films, which coincides with findings from other studies on chitosan- or starch-based films [47,48,49,50]. In contrast, it is observed that this parameter decreases for (bio)PEC films. A lower L* value represents a darker film; notably, the film with a *z* value close to 1 exhibits the lowest value of lightness. We attribute this to the intrinsically heterogeneous microstructure of the (bio)PEC films, which is formed by electrostatic interactions between the polyelectrolytes and efficiently scatters light, thereby reducing its luminosity. Regarding the a* (red–green) and b* (yellow–blue) values, it is observed that the Chit/Starch and (bio)PEC films exhibit negative and positive values for a* and b*, respectively, with certain differences between them. In films based on a mixture of chitosan and native starch, the color depends essentially on the ratio of polymers used due to the absence of strong ionic interactions between biopolymers. The results obtained in this study are consistent with the values reported for the pure biopolymer films, which present a* and b* values of −0.16 and +2.63 for starch films [51] and −2.03 and +11.05 for chitosan films [24,52]. On the other hand, films based on the (bio)PEC exhibited a shift towards green tones (more negative a* values) and yellow tones (more positive b* values). Compared to Chit/Starch films these chromatic alterations can be attributed to the formation of ionic bonds between the amino groups in chitosan and the carboxymethyl groups in CMS. This matrix alters the micro-environment of the inherent chromophores, which are responsible for its yellow coloration in chitosan, leading to an intensification of these tonalities in the (bio)PEC films [52,53].

**Figure 4:**
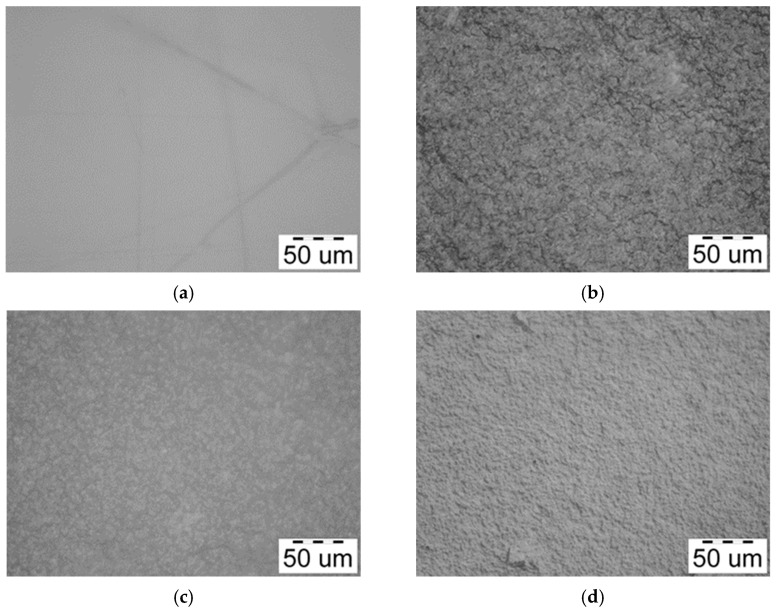
Optical microphotographs of (**a**) Chit/Starch, (**b**) Chit/CMS 1, (**c**) Chit/CMS 2 and (**d**) Chit/CMS 3 films.

Figure 4 shows optical micrographs of films based on a mixture of chitosan and cassava starch, as well as based on (bio)PEC.

Figure 4a shows that the Chit/Starch film does not have a developed morphology, does not contain defects and is transparent to visible light. This corresponds to the high miscibility and compatibility of the biopolymers used, which leads to a completely isotropic structure. On the other hand, the micrographs of films based on polyelectrolyte complex show an irregular grain-like surface. This kind of surface is according to the results from another works [24,48,54]. This irregular structure of the films can be explained as result of the ionic interaction between chitosan and CMS. Additionally, the film formulation Chit/CMS 3 (Figure 4d) with *z* = 0.98 was characterized by the smoothest surface in comparation with other films based on (bio)PEC (Figure 4b,c). We believe that this outcome is attributable to the charge balance achieved between the polyelectrolytes upon the formation of a stoichiometric polyelectrolyte complex at *z* ≈ 1. This specific type of (bio)PEC structures (coacervate particles) formed in the film-forming solutions is insoluble, small, and compact. Consequently, during solvent evaporation in the film formation process, the (bio)PEC particles tend to coagulate and fuse together. This structure in an intrinsically dense and continuous film with a notably smoother surface morphology is formed.

**Figure 5:**
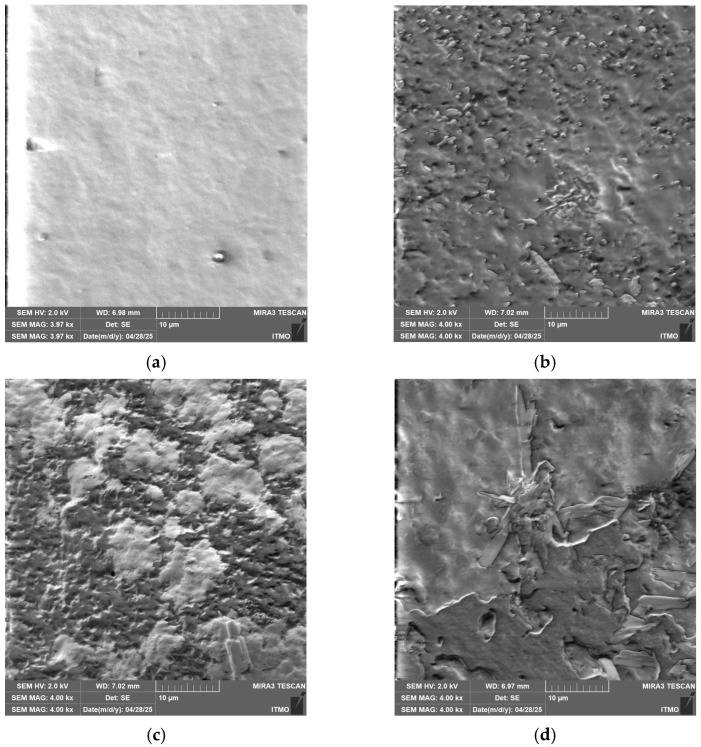
SEM microphotographs of (**a**) Chit/Starch, (**b**) Chit/CMS 1, (**c**) Chit/CMS 2 and (**d**) Chit/CMS 3 films.

Figure 5 shows SEM electron microphotographs of films based on a mixture of chitosan and cassava starch, as well as based on (bio)PEC.

The SEM micrographs, showed in Figure 5, corroborate the previous observations made with optical microscopy. The most uniform microstructure with the lowest pore density is observed in the Chit/Starch films (Figure 5a), which is attributable to the high miscibility of these biopolymers. In contrast, films based on (bio)PEC shows a decrease in surface homogeneity and is observed with the formation of a grain-like surface, a consequence of the electrostatic interactions between the polyelectrolytes. Similar findings were reported by Pilicheva B., Uzunova Y. and Marudova M. [55] in polyelectrolyte films based on chitosan and casein, where the interpenetrating layers were also attributed to polyelectrolytic interactions. The characteristic irregular surface of films based on (bio)PEC originates from the mechanism of their formation. The process occurs in several stages: in the first stage, the polyelectrolyte complex forms, giving rise to primary (bio)PEC structures. In a second stage, these structures associate through intermolecular interactions (hydrogen bonds, ionic bonds, and Van der Waals forces) to form of complex coacervates of (bio)PEC. The final stage of solvent evaporation consolidates this architecture where the random arrangement and packing of polymeric aggregates result in a grain-like surface.

### 3.3. Chemical Composition of Films

**Figure 6:**
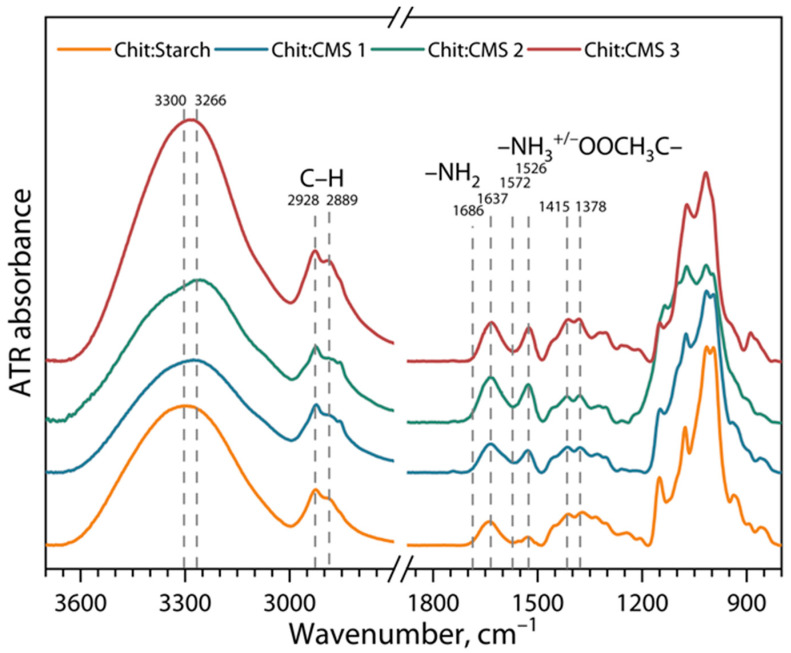
ATR-FTIR spectra of Chit/Starch, Chit/CMS 1, Chit/CMS 2 and Chit/CMS 3 films.

Figure 6 shows the ATR-FTIR spectra of films based on (bio)PEC with different types of CMS, and films based on chitosan and cassava starch.

In general, the spectra of the (bio)PEC-based films shows similarities among themselves, however new bands and more intense peaks are shown in comparison to the Chit/Starch film. Firstly, the bands with center between 3300 and 3266 cm^−1^ become narrower with increasing *z* value. The observed behavior is consistent with redistribution of free side hydroxyls in polysaccharides during the formation of a film with a different type of CMS, as well as due to electrostatic interactions [24,56,57,58]. Furthermore, it can be seen that the stretching vibrations of C-H (2928 and 2889 cm^−1^) become more intense due to the presence of CH_2_ groups in the CMS [59,60]. Small changes in the width of the center strips 1637 cm^−1^ for (bio)PEC-based films are attributed to the stretching vibration of the C=O presented in CMS. Additionally, the bands at 1572 cm^−1^ and 1526 cm^−1^ correspond, respectively, to the NH_2_ and NH_3_ groups presented in chitosan. Hydrogen bonds and ionic interactions between chitosan and CMS are manifested in bands between 1686 cm and ^1^ and 1572 cm^−1^, and for films based on (bio)PEC these interactions are more intense than for films based on a mixture of chitosan and native starch [30,61].

### 3.4. Thermal Stability of Films

**Figure 7:**
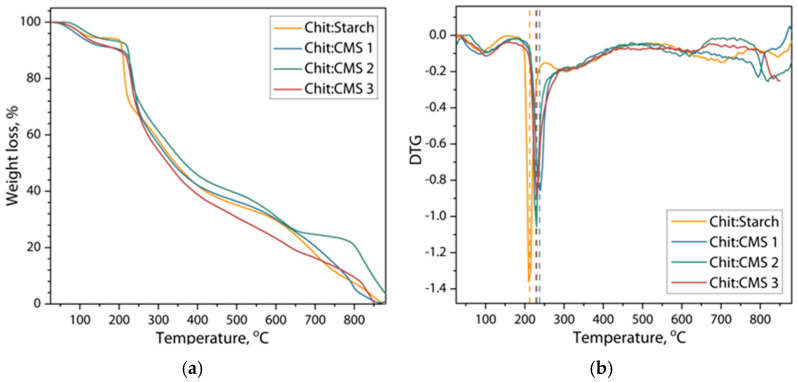
(**a**) Thermograms and (**b**) derivative thermogravimetric curves of weight loss of Chit/Starch, Chit/CMS 1, Chit/CMS 2 and Chit/CMS 3 films.

Figure 7 shows thermograms and the derivate thermogravimetric curves of weight loss of obtained films based on Chit/Starch and based on (bio)PEC Chit/CMS.

From the thermograms it can be seen that the first stage of weight loss of the samples lies in the range of 25 to 140 °C and refers to the residual evaporation of moisture from the films. At the same time, films based on (bio)PEC show a slightly higher moisture content. This effect could be attributed to the presence of carboxymethyl groups in the CMS which increased the hydrophilicity of the CMS and formed a water-trapping network [62]. The second stage of weight loss observed between 200 and 300 °C in both types of films corresponds to saccharide degradation, depolymerization, thermal and oxidative decomposition of biopolymers [29]. The center of this stage is located at a temperature of 210 °C with a weight loss of about 12% for a Chit/Starch film. However, for a film based on (bio)PEC this peak is already about 230 °C. This observation indirectly confirms the hypothesis of the formation of a large number of ionic bonds in films based on (bio)PEC is compared to films where native cassava starch is used, which leads to an increase in the threshold amount of heat for their destruction, thereby increasing the thermal stability of the films. Some works corroborate the fact that an increase in molecular interactions allows ] higher destruction temperatures in polymeric compounds [27,44,45]. For example, Maciel V., Yoshida C., and Franco T. [27] studied the thermal degradation of films formed by chitosan and pectin PEC, observing that the structural decomposition starts above 250 °C because of the complexation of chitosan and pectin. In another study [44] complexes based on polyethylenimine (PEI) and poly (4-styrenesulfonic acid) (PSS) were obtained, and a higher destruction temperature, due to the ionic interaction between the animo and sulphonate groups was shown.

Kinetic analysis was carried out according to the Reich–Fuoss method (Equation (5)) for the major degradation process in each films sample from the thermograms and derivate thermogravimetric curves [63,64]

(5) $$∆ln\left(\frac{dW}{dT}\right)=n\cdot \ln\left(\frac{A}{\beta }\right)+\frac{Ea}{R}\left[\frac{W_{0}}{-\frac{dW_{0}}{dT}\cdot T_{0}^{2}}\cdot lnW-\frac{1}{T}\right],$$

where *W* is the converted weight fraction of the sample; *T* is the temperature; *dW*/*dT* is the weight loss rate of the sample; *n* is the reaction order; *A* is the pre-exponential factor; β  is the heating rate; *Ea* is the energy of activation; *R* is the universal gas constant; *W*_0_, *dW*_0_/*dT* and *T*_0_ are the weight loss, weight loss rate and temperature corresponding to the point of inflection of the derivate thermogravimetric curve.

**Figure 8:**
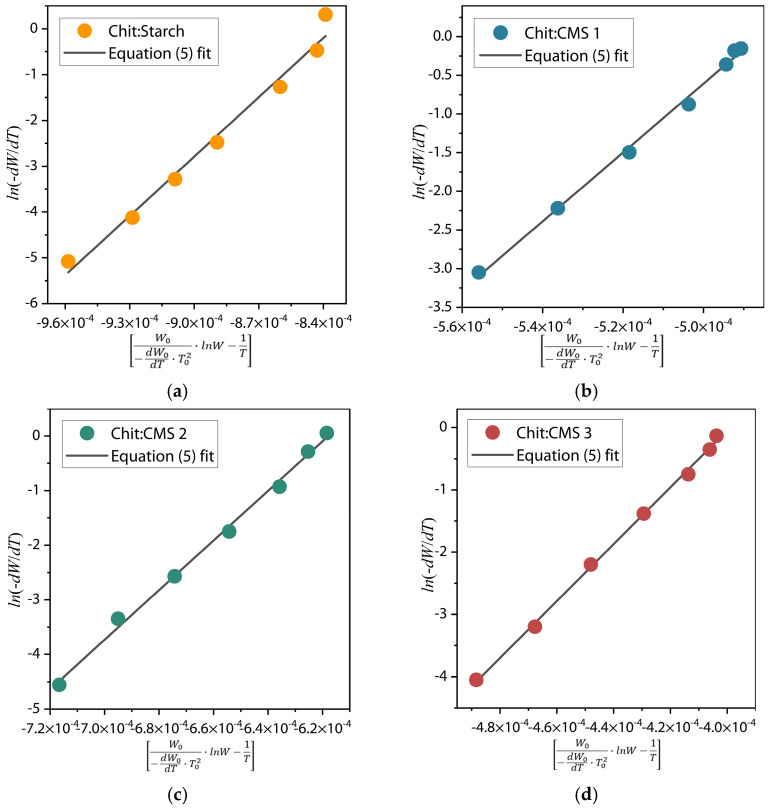
$ln\left(-\frac{dW}{dT}\right)$ in dependence of $\left[\frac{W_{0}}{-\frac{dW_{0}}{dT}\cdot T_{0}^{2}}\cdot lnW-\frac{1}{T}\right]$ for (**a**) Chit/Starch, (**b**) Chit/CMS 1, (**c**) Chit/CMS 2 and (**d**) Chit/CMS 3 films.

The energy of activation (*Ea*) was calculated form the slope (*Ea*/*R*) of the plot $ln\left(-\frac{dW}{dT}\right)$ in dependence of $\left[\frac{W_{0}}{-\frac{dW_{0}}{dT}\cdot T_{0}^{2}}\cdot lnW-\frac{1}{T}\right]$ near the value *T* = *T*_0_ (Figure 8).

The values of imaginary order of the decomposition reaction (*n*) and imaginary order of the decomposition reaction (*k*) were calculated according to Equations (6) and (7) [63,64]

(6) $$n=\frac{{Ea\cdot W}_{0}}{R\cdot T_{0}^{2}\cdot \frac{dW_{0}}{dT}},$$

(7) $$k=-\frac{dW_{0}}{dT}\cdot \frac{\beta }{W_{0}^{n}}$$

Table 2 represents the fitting parameters of Reich–Fuoss model (Equation (5)) of each film sample.

From Table 2, it can be observed that the highest activation energy (*Ea*) corresponds to the Chit/CMS sample. This demonstrates that the formation of (bio)PEC structures confers significantly superior thermal stability compared to films based on the mixture of chitosan and native starch, as a result of the ionic bonds formed. Furthermore, a direct correlation between the degree of substitution (DS) of CMS and the material’s thermal stability is evident, where a higher DS of CMS (Chit/CMS 3) resulted in the highest *Ea* (379.70 kJ/mol). This increase is attributed to a higher density of ionic crosslinking between the carboxymethyl groups of CMS and the amino groups of chitosan. In (bio)PEC films, the high *n* value may be due to the highly cooperative and effective electrostatic interactions between the polyelectrolytes. This cooperative nature results in highly effective binding constants, driving the formation of stable complexes, as shown in the turbidimetric titration results. Likewise, the concomitant decrease in the pre-exponential factor (*k*) with increasing DS confirms the presence of a kinetic compensation effect.

### 3.5. Results of Differential Scanning Calorimetry (DSC) of Films

**Figure 9:**
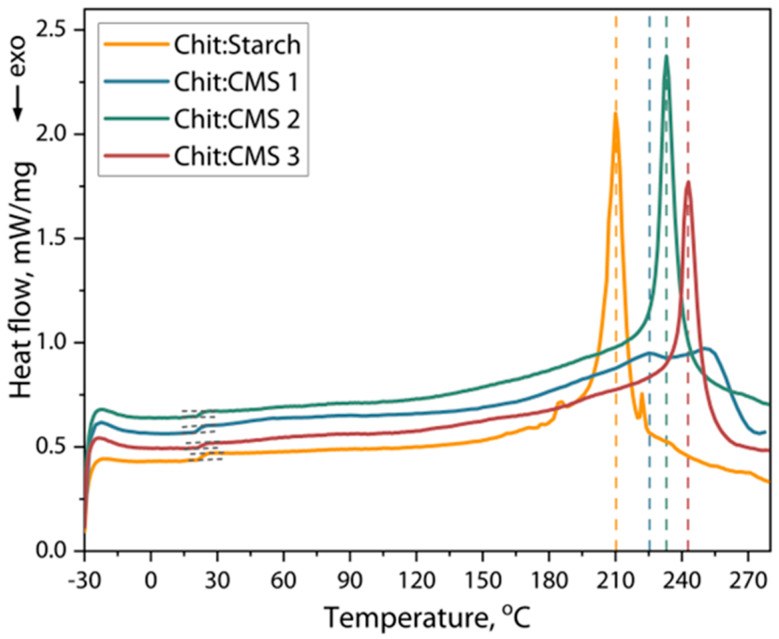
DSC heating curves of Chit/Starch, Chit/CMS 1, Chit/CMS 2 and Chit/CMS 3 films.

Figure 9 shows the DSC curves of obtained films based on Chit/Starch and based on (bio)PEC of Chit/CMS.

The glass transition temperature (*Tg*), degradation temperature (*Td*) and enthalpy changes (Δ*H*) of the samples were calculated from Figure 9, and the results are presented in Table 3.

The thermal analysis of the films, as shown in Figure 9, reveals DSC curves with slight differences between the samples. Two main thermal events were identified in all samples: a glass transition in the range of 20 to 23 °C, and a significant endothermic event at temperatures above 180 °C, associated with softening of the polymer matrix and the onset of thermal decomposition. The *Tg* values, shown in Table 3, indicate that there are no differences in the activation of segmental mobility for all the samples studied. We believe that this behavior is due to the effect of the presence of plasticizer in all films, which has a more pronounced effect on the segmental mobility of chitosan, starch and CMS chains. The presence of plasticizer increases the mobility of unlimited chain segments, partially compensating for the expected effect of increased rigidity of macromolecular chains due to ion crosslinking [24,65].

Conversely, the temperature associated with the main endothermic event (*Td*) was consistently lower for the Chit/Starch films compared to the (bio)PEC films. This result is consistent with expectations, as the network cohesively bound by ionic bonds in the polyelectrolyte complex confers greater thermal stability to the material. Consequently, a higher energy input (higher temperature) is required to initiate the breakdown of this structure and the degradation processes. This interpretation is supported by the enthalpy (Δ*H*) values of this event, which were 17–25% higher for the (bio)PEC films in comparation with chitosan and starch films. The increase in Δ*H* indicates that greater energy is required to disrupt the (bio)PEC structure, which is consistent with the higher density of intermolecular ionic bonds that must be overcome during the thermal process.

### 3.6. Mechanical Properties of Films

**Figure 10:**
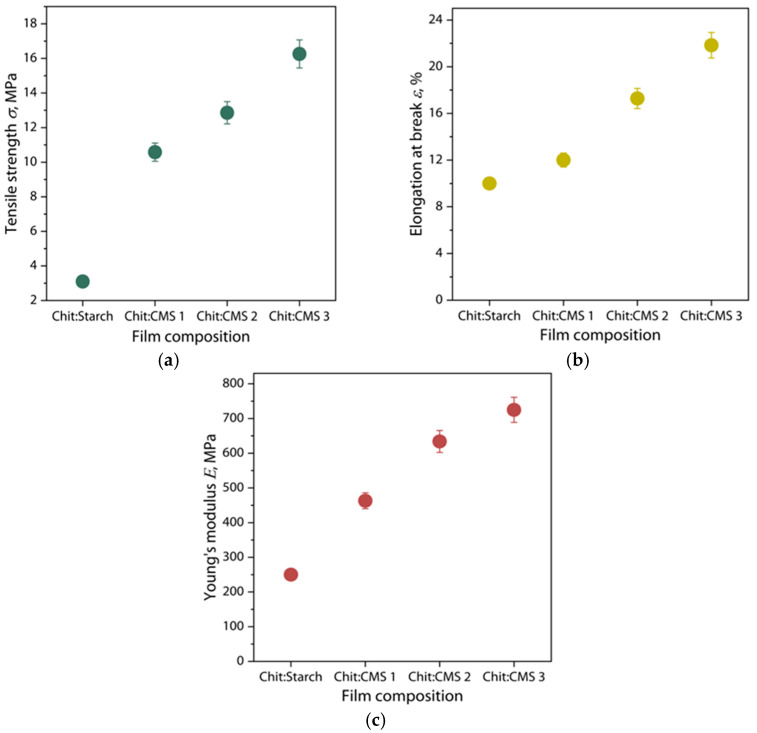
(**a**) Tensile strength, (**b**) elongation at break and (**c**) Young’s modulus of Chit/Starch, Chit/CMS 1, Chit/CMS 2 and Chit/CMS 3 films.

The mechanical properties of the Chit/Starch and Chit/CMS films are shown in Figure 10.

The results demonstrate that the tensile strength, Young’s modulus and elongation at break are higher in the samples obtained from (bio)PEC dispersions (Figure 10). This is mainly due to the presence of ionic bonds between the carboxymethyl groups of CMS and the amino groups of chitosan. These electrostatic interactions hold polymer chains together, resulting in enhanced cohesion between the polymeric chains, and create a more compact and durable structure. Some studies have confirmed this improvement in the mechanical properties of (bio)PEC films [66,67,68]. For example, Yin Y. et al. [66] studied (bio)PEC films formed by chitosan and gelatin, observing a maximum increase in the tensile strength with a chitosan content of 0.5, which corresponds to optimum compatibility between chitosan and gelatin with a decreasing of the crystallinity of film. Also, in another work [69] the mechanical properties of polyelectrolyte complex films obtained on the basis of Na-carboxymethylcellulose (Na-CMC) and polyacrylamide (PAA) were studied. The authors demonstrated that an increase in PAA content up to 60% of the total PEC content allows a 7-fold increase in tensile strength and an 80-fold increase in elastic modulus with respect to Na-CMC films. However, higher amounts of PAA lead to a decrease in the mechanical properties. This is due to the fact that with the increase in PAA, the interaction between the components is increased, increasing the frequency of crosslinking between the constituent elements of the PEC. On the other hand, a higher increase in PAA, more than 60%, leads to the formation of a heterogeneous structure of the films, decreasing their mechanical properties [69]. The low mechanical properties of the samples obtained from the mixture of chitosan and native starch is due to the fact of limited physical interaction between the biopolymer chains. In these films, the predominant interactions are weak hydrogen bonds between the hydroxyl groups of starch and the amino groups of chitosan, which limits the formation of a robust three-dimensional network and, consequently, their limited mechanical properties [70,71].

Additionally, it is observed that the increase in the DS of used CMS and, respectively, increase in *z* value in (bio)PEC leads to a significant increase in the mechanical properties. This observation indicates a strong dependence of the strength and elasticity of the resulting film structure on the ratio of ionized polyelectrolyte groups during the formation of complex (bio)PEC coacervates and the subsequent self-assembly of the polymer matrix from these dispersions. It is logical to assume that, as the value of *z* approaches 1, the density of ionic crosslinks in the final material structure increases, leading to the formation of a stronger reinforcing network compared to a material without polyelectrolyte interactions between the polymers [36,69,72].

### 3.7. Solubility of Films in Liquids

**Figure 11:**
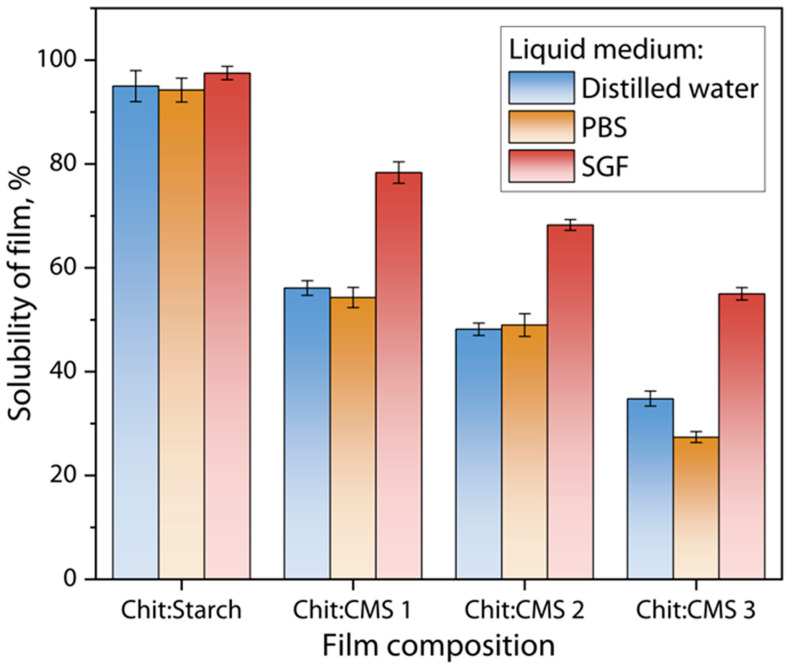
Solubility of Chit/Starch, Chit/CMS 1, Chit/CMS 2 and Chit/CMS 3 films in distilled water (pH = 6), SGF solution (pH = 1.2), and PBS solution (pH = 7.4) at 37 °C.

Film solubility tests were performed gravimetrically by keeping samples in liquid media such as distilled water, PBS and SGF solutions. In general, the stability of the films depends mainly on the number of free ionized groups on the surface of the (bio)PEC films; the amount of ionic bonds and hydrogen bonds formed; and the ionic strength and pH of the medium. The solubility of films in different liquids at 37 °C is shown in Figure 11.

Figure 11 shows that the solubility of the films in the SGF solution at pH = 1.2 is higher compared to the solubility in water and in the PBS solution. These differences can be attributed mainly to the nature of the molecular interactions and the resulting structure of each type of film [73]. For the Chit/Starch film the solubility is the highest (97.5 ± 1.3%) because the polymer matrix is formed by hydrogen bonds, which are weak and easily competed by the water molecules present in the aqueous medium at low pH. In contrast, the solubility of the films with compositions of Chit/CMS 1, Chit/CMS 2 and Chit/CMS 3 were 78.35 ± 1.3%, 68.26 ± 1.04% and 54.99 ± 1.20%, respectively. It is evident that for Chit/CMS (bio)PEC films the solubility was lower and decreased with the increase in ratio of ionized functional groups of chitosan and CMS (*z*). This behavior stems from the alteration in the ionic interactions that stabilize the structure of the material. The stability of the PEC arises from electrostatic attractions between the protonated amino groups of chitosan (-NH_3_^+^) and the ionized carboxylate groups of CMS (-COO^−^), and when *z* is near to 1 there are few free chargers in the biopolymer matrix. Additionally, in the highly acidic medium, like SGF, these carboxylate groups undergo extensive protonation, shifting the equilibrium towards the uncharged carboxylic acid form (-COOH). Similarly, in acidic media, the decrease in ionic interactions in the polymeric complex generates a less compact structure, which facilitates water penetration and causes a greater hydration and solubility of the film. Consequently, the attractive ionic forces between the polyelectrolytes are significantly diminished, compromising the integrity of the complex [61].

For the water and PBS solution, the trend of higher solubility for Chit/Starch films, compared to (bio)PEC films, is also consistent within both mediums. For the Chit/Starch films, solubility in both media varies depending on the proportion of polymers in the mixture and generally exhibits higher values. At pH near to the neutral, this heightened solubility is attributable to the hydrophilic nature of the biopolymeric matrix formed, due to the amino groups of chitosan, which facilitates the interaction with water molecules, increasing the moisture absorption and, therefore, the solubility of the films [74]. On the other hand, (bio)PEC films Chit/CMS exhibit reduced solubility because ionic network creates a denser and less water-permeable structure than the hydrogen-bonded network of Chit/Starch matrix [28,43]. This phenomenon is well-corroborated in the literature. For instance, a study on furcellaran–chitosan systems found that standalone films had higher solubility than PEC films with an 8:2 ratio [24]. The authors ascribed this to the markedly stiffer structure of the PEC, which limits water accessibility to hydrophilic sites. Shahabazi et al. [48] reported congruent findings upon forming a PEC between chitosan and carrageenan. For this system, the water solubility decreased to 23.3%, because the binding of the hydrophilic chains between the polyelectrolytes led to a relatively stiffer structure, which decreased the accessibility of water to the hydrophilic groups.

### 3.8. Surface Wettability of Films

The water contact angle (CA) related to surface of obtained films are given in Table 4.

The results show that the CA values are significantly higher in films based on (bio)PEC. Angles lower than 90° are associated with hydrophilic surfaces, but for films of Chit/Starch with a large number of free polar groups (hydroxyl, carboxyl and amino) the surface has a high density of charges and a marked affinity for water. In contrast, the presence of ionic bonds between polyelectrolytes in structure of (bio)PEC films produces a partial neutralization of surface charges. This phenomenon reduces the availability of free polar groups, leads to a more compact structure, and lowers the surface energy. Consequently, these changes result in an increased CA and reduced hydrophilicity [75,76].

## 4. Conclusions

In the current work, films based on a (bio)polyelectrolyte complex of chitosan and carboxymethyl starch (CMS) with varying degrees of substitution (DS) were successfully obtained using the casting method. The film-forming solutions were prepared by mixing independent solutions of chitosan and CMS (2.5 wt.% concentration) at a 1:3 *v*/*v* ratio, adjusting the pH to achieve maximum efficiency of (bio)PEC structures formation. Concerning the aspect of the films, the analysis of the color parameters in the CIE L*a*b* system shows that the lightness of the films based on (bio)PEC is reduced compared to the mixture of chitosan and native starch, a phenomenon attributed to increased light scattering (turbidity) from the heterogeneous microstructure of the complexes forming the solution. In addition, the optical and scanning microscopy showed the irregular surface of the films based on (bio)PEC. The formation of (bio)PEC in the film structure was corroborated by ATR-FTIR spectroscopy, that showed changes in the bands between 1686 and 1526 cm^−1^. These changes confirm the interaction between chitosan and CMS. The thermogravimetric analysis showed a higher thermal stability for the (bio)PEC films thanks to the higher structural density, due to the generation of a more compact and organized polymeric network. The results of mechanical tests of (bio)PEC films under tension showed that the (bio)PEC films exhibited significantly higher tensile strength, Young’s modulus and elongation at break than the chitosan/starch films. This improvement is attributed to the strong ionic interactions and hydrogen bonding between the amino and carboxymethyl groups, which improved the cohesion and structural organization of the polymeric network. In terms of solubility, the (bio)PEC films showed reduced solubility in water, PBS and SGF solutions compared to the chitosan/starch films, due to the compact and less water-permeable structure formed by ionic interactions. The properties of films based on (bio)PEC of chitosan and CMS with ratio 1:3 *v*/*v* enhance the potential applications of these materials, particularly in the development of active and durable coatings for the food industry, due to their improved mechanical properties and low solubility in aqueous solutions.

Finally, promising prospects are identified for the improvement in the obtained films. Although films based on (bio)PEC exhibit improvements in their mechanical properties and a reduction in their solubility in aqueous media, a more thorough characterization would allow their potential to be fully evaluated. Studies of water vapor permeability, surface energy, folding resistance and microbial activity can be utilized. Additionally, exploring their application as edible coatings on perishable foods, such as strawberries, through food quality tests that determine their impact on shelf life, would represent a crucial advance for their large-scale implementation.

## Figures and Tables

**Table 1:** Color parameters (L*, a*, and b*) and appearance of films samples.

Film Composition (Values of z)	L*	a*	b*
Chit/Starch	93.32 ± 0.11	−0.30 ± 0.05	2.90 ± 0.09
Chit/CMS 1 (0.30)	71.41 ± 0.35	−1.69 ± 0.03	7.92 ± 0.12
Chit/CMS 2 (0.64)	69.95 ± 0.11	−1.37 ± 0.01	7.92 ± 0.01
Chit/CMS 3 (0.98)	67.71 ± 0.04	−1.67 ± 0.01	7.44 ± 0.06

**Table 2:** Parameters of Reich–Fuoss model (Equations (5)–(7)) for film samples.

Sample	*T*_peak_, °C	*Ea*, kJ/mol	*n*	*k*	*r* ^2^
Chit/Starch	209.88	358.84	11.87	1.23 × 10^−22^	0.984
Chit/CMS 1	239.89	370.16	14.99	5.49 × 10^−28^	0.997
Chit/CMS 2	229.87	378.05	14.01	1.52 × 10^−26^	0.996
Chit/CMS 3	232.79	379.70	16.50	3.37 × 10^−31^	0.998

**Table 3:** Thermal properties of films based on Chit/Starch and (bio)PEC of Chit/CMS.

Sample	*z*	*Tg*, °C	*Td*, °C	Δ*H*, J/g
Chit/Starch	-	23.14	210.02	127.30
Chit/CMS 1	0.30	21.87	226.12	149.70
Chit/CMS 2	0.64	20.48	233.07	172.20
Chit/CMS 3	0.98	22.06	242.70	158.50

**Table 4:** Contact angle of water for films.

Sample	*z*	CA *θ*,°
Chit/Starch	-	75.7 ± 0.8
Chit/CMS 1	0.30	79.9 ± 1.3
Chit/CMS 2	0.64	82.7 ± 0.9
Chit/CMS 3	0.98	85.7 ± 1.2

## Data Availability

The original contributions presented in this study are included in the article. Further inquiries can be directed to the corresponding author.

## Abbreviations

The following abbreviations are used in this manuscript:

Bio(PEC)	(Bio)polyelectrolyte complex
Chit	Chitosan
CMS	Carboxymethyl starch
TPP	Sodium tripolyphosphate
MMT	Montmorillonite
PEMA	Poly(ethylene alt-maleic acid)
PAA	Poly(acrylic acid)
PEI	Polyethylenimine
SGF	Simulated gastric fluid
PBS	Phosphate-buffered saline
z	Ratio of ionized functional groups
n	Number of the samples
σ	Tensile strength
E	Young’s modulus
